# Treatment of Major Depressive Disorder in Pediatric Populations

**DOI:** 10.3390/diseases6020048

**Published:** 2018-06-04

**Authors:** Drew R. Neavin, Jeremiah Joyce, Cosima Swintak

**Affiliations:** 1 Department of Molecular Pharmacology and Experimental Therapeutics, Mayo Clinic Graduate School of Biomedical Sciences, Mayo Clinic, Rochester, MN 55902, USA; Neavin.Drew@mayo.edu; 2 Mayo Clinic School of Medicine, Mayo Clinic, Rochester, MN 55902, USA; Joyce.Jeremiah@mayo.edu; 3 Department of Psychiatry, College of Medicine, Rochester, MN 55902, USA

**Keywords:** major depressive disorder, pediatrics, treatment

## Abstract

Major depressive disorder (MDD) is a severe illness that afflicts about 16.6% of people over their lifetime. MDD is highly correlated with suicidality, and often first presents in adolescence. Unfortunately, many pediatric patients suffering from MDD go undiagnosed, and current evidence-based treatment options in the U.S. are limited to psychotherapy and two selective serotonin reuptake inhibitors approved by the United States Food and Drug Administration. Molecular mechanisms have been shown to play a role in MDD pathogenesis, progression, and response to medication, yet few studies have explored the role of these pathways in pediatric MDD. In this review, we outline the gravity and importance of MDD in pediatric patients, some challenges in diagnosis and treatment, current treatments available for pediatric patients, and research to investigate differences between pediatric and adult MDD. We hope that this review will provide an outline of the current understanding and treatment of MDD in pediatric patients, and provide thoughtful insights for future work that could advance our understanding of MDD in pediatric populations, and also identify new therapeutic strategies.

## 1. Introduction

Major depressive disorder (MDD)—the leading cause of disability worldwide [1]—is characterized by depressed mood, low self-worth, and altered sleep and appetite [1]. MDD affects people of a wide range of ages, and can be recurrent throughout an individual’s lifetime. MDD often first presents in adolescence [2,3], and the onset of depression is most frequent between the ages of 10 and 18 [2,3]. The overall lifetime prevalence of MDD is estimated at 16.6% [4], with a cumulative 9.5% prevalence in pediatric patients by the age of 16 years [5]. Further, MDD is the leading cause of morbidity in adolescents [6], and adolescent depression is highly correlated with longer and more recurrent depression in adulthood [2,7]. MDD is also highly correlated with suicidality, which is the third highest cause of death in adolescents worldwide [6]. The gravity of this illness cannot be overstated, since it affects all ages, including pediatric populations, and can ultimately result in premature death.

The prevalence of pediatric MDD increases with age [8,9] with a spike in MDD diagnosis following puberty. This is thought to be due most likely to physical changes, which include increased hormonal production and signaling, as well as social environmental changes, including psychosocial pressures and increased responsibilities [10]. Psychosocial risk factors in early life are important contributors to the pathogenesis of many diseases. Ongoing research has linked adverse childhood experiences (ACEs) to early death and increased incidence of diseases, including alcoholism, ischemic heart disease, and depression [11]. ACEs are grouped into categories of abuse (physical, sexual, or emotional), household challenges (such as incarceration of a family member or parental separation or divorce), and neglect (either physical or emotional). ACEs have been correlated with the increased prevalence of depressive disorders later in life (odds ratio = 2.7 for women and odds ratio = 2.5 for men). Further, there is a strong dose-dependent relationship between the number of ACEs and the risk of developing a depressive disorder [12]. Gender differences in the diagnosis of MDD become apparent following puberty, with about a two-fold higher rate of diagnosis in girls than in boys [13]. Other risk factors for MDD include a familial history of MDD, environmental exposures, chronic inflammation, diet, and exercise [14]. 

The molecular pathways that lead to the pathogenesis of MDD are not fully understood. There is growing evidence that a number of different pathways may play a role in the pathogenesis of MDD, including inflammation, immune response, monoamine neurotransmission, the hypothalamic–pituitary–adrenal (HPA) axis, and the gut–brain axis [15,16]. Notably, the environmental and psychosocial factors mentioned above have also been shown to impact these molecular pathways [14,17]. Specifically, exercise and dietary modulation have been associated with altered inflammatory modulators, variation in monoamine neurotransmitters, and differences in the HPA axis—all pathways that can play a role in MDD pathogenesis [14]. The pathways that may contribute to MDD pathogenesis are discussed in more detail in section 4 (Biomarkers and Pathways of Pediatric MDD). Many of the antidepressants currently in use target monoamine neurotransmission but are not effective in 20–50% of patients [18,19], indicating that these patients may be suffering from symptoms of MDD caused by different molecular mechanisms.

## 2. Diagnosis of Pediatric MDD

While most of the main features that characterize MDD in adults are the same or similar in pediatric patients, there are some key differences (Table 1) [13]. Depressed mood, alterations in sleep, and changes in eating patterns are some of the main symptoms that are seen in both adult and pediatric MDD patients. Symptoms that are more prevalent in pediatric MDD include social withdrawal, irritability, and weeping. Early indicators of depression in pediatric patients can include decreased interest in hobbies and other leisure activities, altered interest in social interactions, and decreased academic achievement [17,20]. Unfortunately, many of these symptoms can be overlooked or mistaken for ‘typical’ teenage behavior, especially since mood swings often coincide with puberty—the age of onset of depression in many patients. In addition, pediatric patients may not yet have developed an adequate emotional vocabulary, and/or may lack emotional insight, making it difficult for them to communicate their emotional states. Together, these characteristics make MDD challenging to accurately diagnose in adolescent populations [13].

Because of the aforementioned complications of symptom identification, and because many adolescents are simply not screened for depression, many pediatric patients suffering from MDD go undiagnosed. Several screening questionnaires are utilized in pediatric populations. Two specific examples include the Patient Health Questionnaire for Adolescents and the Beck Depression Inventory-Primary Care Version, both of which focus on assessing the severity and frequency of symptoms related to MDD. These can be helpful in making the diagnosis of MDD in adolescents and children [21], especially when understood in the broader context of a clinical presentation. When five or more of the main symptoms of MDD (Table 1) are present (with either depressed mood or decreased interest/pleasure), a patient can be diagnosed with MDD. However, the symptoms must significantly impact daily activities, and should not be due to a medication or other condition. In addition, a previous manic episode is an indication of a separate medical condition, such as bipolar depression [22].

Recent reviews of practices for diagnosis and treatment of pediatric MDD have recommended yearly screening for MDD in all adolescent patients (ages 12–17) in order to decrease the number of undiagnosed pediatric patients, and to provide symptom-relieving treatment sooner. A clear benefit of the universal screening of depression in children (younger than age 12) has not yet been established according to current Guidelines for Adolescent Depression in Primary Care and U.S. Preventive Services Task Force guidelines [21,23].

## 3. Current Pediatric MDD Treatment

Currently, psychotherapy and two selective serotonin reuptake inhibitors are approved for the treatment of pediatric MDD in the U.S. These treatments, the ages for which these treatment options are indicated, and potential concerns for use in pediatric populations are subsequently outlined, and are summarized in Table 2.

### 3.1. First-Line Therapeutic Approaches for Pediatric MDD Treatment

Clearly, MDD is a serious illness that carries a significant burden at both the individual and population levels, yet therapies for pediatric patients are limited [23]. Psychotherapy—interpersonal treatment based on psychological principles—is often regarded as the first-line therapeutic strategy for MDD in pediatric patients. In this population, two of the most frequently used and best studied psychotherapeutic approaches are Cognitive Behavioral Therapy (CBT) and Interpersonal Therapy (IPT). These therapies can be effectively administered in a variety of settings (inpatient or outpatient, group-based or individual) by trained professionals, including psychologists, psychiatrists, social workers, licensed therapists, and family therapists [24,25]. Unfortunately, the current supply of pediatric-trained professionals does not adequately meet the demand of depressed pediatric patients [26]. 

CBT is based upon the well-understood interactions between thoughts, behaviors, and feelings. Patients are taught to identify maladaptive patterns of reasoning, known as cognitive distortions, in order to replace them with more constructive thoughts, thereby lessening emotional distress and self-destructive behaviors. This is done through a variety of interventions, including exposure therapy, mindfulness, motivational self-talk, and biofeedback. IPT, which focuses on building relationship and communication skills, is relatively less studied, but has also been used for the treatment of pediatric MDD [24,25,27].

A recent meta-analysis of 42 randomized clinical trials that studied the effect of psychotherapy on depressed adolescents (aged 13–24) and children (aged less than 13) concluded that both CBT and IPT had statistically significant positive effects for adolescents, and both modalities were deemed as “well-established” for the treatment of MDD in that population. Unfortunately, CBT was deemed only “possibly efficacious” for children, with more investigation required. No randomized clinical trials investigating IPT in children were reviewed. Differences in effectiveness between the age groups may be related to the developmentally appropriate capacity for self-awareness, introspection, and self-improvement. The study raised concerns regarding the lengthy treatment course, natural remission, limited durability of treatment over time, and subjective nature of control therapies. In addition, it can be difficult to separate the psychotherapy component of CBT and IPT from the response to the therapeutic milieu of the clinical setting in pediatric patients [27].

### 3.2. Pharmacological Therapeutics for Pediatric MDD Treatment

In addition to non-pharmacological psychotherapy, there are two United States Food and Drug Administration (FDA)-approved selective serotonin reuptake inhibitors (SSRIs) for the treatment of adolescent MDD: fluoxetine (ages 8–18) [28,29] and escitalopram (ages 12–17) [30]. SSRIs have shown mixed efficacy in pediatric MDD clinical trials, with some trials reporting a larger reduction in depression symptoms and greater prevalence of remission as compared to placebo [31]. Even though these SSRIs are FDA-approved for pediatric MDD patients, fluoxetine and escitalopram appear to be more effective in adult populations than in pediatric populations [32]. 

Further, the FDA issued a black box warning on SSRIs [29,33,34] based on their association with a small increase in suicidality in adolescent patients, even though there was no reported effect on suicidality in adults [29,32,35,36,37]. In addition, SSRIs have been associated with increased agitation and hostility [38]. However, these findings have not been consistent across all studies. It is important to note that suicide risk is already increased in pediatric MDD patients, and the risk of not treating pediatric MDD patients who require treatment may outweigh the potentially small increased risk of suicide attempt with SSRI treatment on a population level [37,39,40]. Given the evidence for a potential increase in suicide risk during SSRI therapy, the FDA recommends that pediatric MDD patients treated with fluoxetine or escitalopram be monitored on a weekly basis for the first month and then biweekly for another month, in order to help alleviate any increased risk of suicide that may be incurred by fluoxetine treatment [20,28].

The Treatment for Adolescents with Depression Study (TADS)—a clinical trial that used placebo, fluoxetine, and CBT—indicated that the combination of CBT and fluoxetine resulted in the best treatment response in pediatric patients with depression [17]. Suicide attempts and completed suicides were decreased by the combination therapy when compared to fluoxetine alone, indicating that CBT might be capable of alleviating any potential increased risk of suicide with fluoxetine treatment. However, other studies have not demonstrated effect sizes as large as the TADS study. Potential variation may be due to the study population, time at which phenotypes were measured, polygenic variation, and heterogeneity in both the diagnostic and molecular pathways that contribute to the pathophysiology of MDD in pediatric patients, as well as heterogeneity in data collection [28]. Further, not all studies measured drug levels in patients, and, therefore, variation in adherence to medication could also contribute to variation in clinical trial results [20].

Other medications that are used to treat adults with MDD are not recommended for treatment of pediatric MDD because of serious adverse events, or because there is no evidence of relief from MDD symptoms. Clinical trials for tricyclic antidepressants (TCAs) indicate that they are not efficacious in children with MDD, but may have some efficacy in adolescent MDD patients [7]. However, clinical trial data also indicate that TCAs may cause cardiac adverse events in pediatric populations, making them contraindicated for pediatric MDD patients [28]. 

The use of antidepressants in depressed patients also poses a risk of excessive mood-elevation to the point of mania/hypomania. This may represent a direct pharmacological effect, or may indicate mood-switching from an unrecognized bipolar disorder diagnosis. In juveniles, this risk may be as high as 9.33% [41]. This poses a diagnostic and therapeutic dilemma, as individuals eventually diagnosed with bipolar disorder commonly present with depressive polarity at onset [42]. Therefore, care must be taken when initiating pharmacologically-based antidepressant treatment in pediatric MDD patients.

There has been no change in the FDA recommendation for pediatric MDD treatment over nearly the past decade. Medications with potential efficacy in adult patients have not demonstrated efficacy in pediatric populations, or have not been used in trials with pediatric patients. Furthermore, medications that are generally safe and efficacious for adults who suffer from MDD may not be safe or effective for children and/or adolescents. 

## 4. Biomarkers and Pathways of Pediatric MDD

MDD is a complex, heterogeneous disease, and the pathophysiology of this disease is not fully understood. Although studies have identified associations between a variety of biomarkers (serotonin, BDNF, cortisol) and depression symptoms and/or clinical outcomes, none have demonstrated sufficient evidence to progress to use in clinical settings [13]. Therefore, there are currently no clinical biomarkers that can be used to diagnose MDD, subclassify patients into potential subgroups, select the best medication, or predict response. The lack of biological markers has made the study of MDD challenging. However, many molecular pathways have been implicated in depression. Amongst others, these include inflammation, altered monoamine neurotransmission, altered neurotrophic factor signaling, and increased HPA signaling [13]. The exact role of any of these systems has not yet been fully elucidated in either adult or pediatric MDD populations. Evidence suggests that many of the biomarkers identified in adult MDD may also be relevant to pediatric MDD [43]. Many of these pathways are still maturing during the course of development, and this may contribute to the considerable differences noted in the response to therapeutics between pediatric and adult MDD patients.

### 4.1. Monoamine Neurotransmission

Altered monoamine neurotransmission has long been thought to contribute to depression, and many pharmacologically-based therapeutics for MDD have targeted monoamine neurotransmission (examples include SSRIs, monoamine inhibitors, norepinephrine reuptake inhibitors, TCAs, and selective norepinephrine reuptake inhibitors). Monoamine-targeting antidepressants have resulted in altered monoamine levels in adult patients, and while altered monoamine levels can be measured within days of initiating monoamine-targeting therapeutics, the antidepressant effects may not be apparent for weeks or even months [44]. This indicates that pathway activation downstream of monoamine receptors may be required for effective antidepressant response [8]. In addition, monoamine pathway transporters and receptors continue to mature during adolescence, with the serotonergic system maturing faster than other monoaminergic systems. This observation may explain, in part, why SSRIs, but not TCAs, are efficacious in pediatric patients, even though both are efficacious in adult MDD populations [8].

### 4.2. Stress and the HPA Axis

The HPA axis has been linked to response to stress, and the immune response. In turn, hyperactivity of the HPA axis has been linked to symptoms of MDD. Dysregulation of the HPA axis has been observed in many studies of both adults with MDD and pediatric MDD patients, although the effect sizes appear to be smaller in the pediatric group. However, there is evidence for increasing activation of the HPA axis in older pediatric patients. Therefore, the HPA axis may become more relevant with the increase in age of pediatric MDD patients [45,46,47]. 

However, hormones have been shown to play a role in the activation of the stress response, and have also been shown to play a role in the incidence of post-pubertal MDD. The approximately 2-fold higher rate of the prevalence of MDD in post-pubertal girls than in post-pubertal boys has been postulated to be due, at least partially, to the role of estrogen in stress responses in the prefrontal cortex, as delineated in animal models [48].

### 4.3. Inflammation and Immune Response

Differences in the role of the immune and/or inflammatory systems in pediatric versus adult MDD patients, and the differences in responses to antidepressant medications are not fully understood. Proinflammatory markers have been shown to be elevated in both adult and pediatric MDD patients when compared to healthy controls. Specifically, IL-6, CRP, IL-1β, and IFN-γ have been shown to be elevated in pediatric MDD patients when compared to healthy controls [49]. In adult populations, the proinflammatory molecules IL-6, TNF-α, and IFN-γ, amongst others, were significantly altered in adults with MDD [50]. Mechanisms by which the immune and inflammatory systems may be overactive include an evolutionary advantage during ‘fight or flight’ situations, or the microbiome–host interaction [51]. Specifically, a stressful situation that activates a ‘fight or flight’ response results in increased heart rate, blood pressure, catecholamines, and cortisol, as well as increased inflammatory activation, which can be measured by increased circulating proinflammatory markers. In addition, dysregulation of the microbiome–host interaction can lead to immune response and inflammatory activation, which is also marked by increased circulating proinflammatory markers. This immune activation may then lead to direct regulation of monoamine pathways through the regulation of expression of metabolizing enzymes, or by stimulating the HPA axis [45].

### 4.4. Neurotrophic Factors

Neurotrophic factors are known to play important roles in the regulation of neurogenesis, neuronal growth, and differentiation. In addition, specific neurotrophic factors (brain derived neurotrophic factor (BDNF), nerve growth factor (NGF), neurotrophin-3 (NT-3), and glial cell line derived neurotrophic factor (GDNF)) have demonstrated differential levels between adolescent MDD patients and healthy adolescent controls. Those neurotrophic factors were also differentially expressed after antidepressant treatment in adults, but have not been studied in pediatric populations [52]. These neurotrophic factors have also been associated with inflammation and autoimmune demyelination, indicating that they could impact MDD symptomatology through various pathways [53].

### 4.5. Metabolizing Enzymes

Another factor potentially impacting antidepressant efficacy in pediatric populations is the reduced expression of the cytochrome P450 enzymes during adolescence. These enzymes are responsible for metabolizing many pharmacological agents including escitalopram and fluoxetine, and their lower expression can have a significant impact on the efficacy of SSRI medications in pediatric populations. Few preclinical studies have used pediatric MDD models; however, studies in pediatric mouse models demonstrated that SSRIs such as escitalopram were effective at alleviating depression-like symptoms, while TCAs were incapable of alleviating those symptoms, mirroring the effectiveness profile of antidepressants in pediatric MDD patients. Therefore, pediatric mouse models may provide a promising platform for preclinical therapeutic testing of antidepressants [8].

### 4.6. Pathway Interactions

Clearly, these pathways do not act independently and have complex interactions with one another, which may provide some explanation for the heterogeneous nature of MDD and response to therapeutics in both adult and pediatric patients. The development and maturation of these pathways in pediatric patients may explain the altered response patterns to pharmacological agents in pediatric MDD patients, as compared to adult populations. In addition, the maturation of these pathways is crucial for normal development, and, therefore, targeting and altering these pathways could be detrimental to development in pediatric MDD patients. This may contribute to the relatively slow development of new pharmacological agents for the treatment of MDD in pediatric patients.

## 5. Discussion and Conclusions

This review has outlined the current understanding of pediatric MDD and the therapeutic options that are available for treatment. Even though MDD is the leading cause of disability worldwide [1] and the leading cause of morbidity in adolescent populations [6], there are only a few evidence-based treatment options for pediatric patients, including psychotherapy and two FDA-approved pharmacological agents (fluoxetine and escitalopram). It is clear that early effective treatment of pediatric MDD patients can minimize long-term effects [13]. However, only about 60% of pediatric MDD patients respond to fluoxetine [17], and there may be a potential increased risk of suicide attempts and suicides carried out in pediatric patients treated with SSRIs [29,32,35,36]. However, suicidality is a symptom of pediatric MDD, and the potentially small increase in suicide attempt risk may not outweigh the benefit of treatment on a population level [37,39,40]. Due to challenges in effectively diagnosing pediatric patients with MDD, screening all pediatric patients may provide more comprehensive coverage of pediatric patients who require treatment [13]. However, there has been a long-standing shortage of professionals who are trained to screen for and diagnose pediatric MDD, making this a currently unrealistic goal [26].

Of note, there have been many advances in the understanding of the molecular mechanisms and treatment of many psychiatric disorders, many of which first present during adolescence, including bipolar depression and schizophrenia. However, there have been fewer advances in the discovery of genetic contributors and biomarkers for MDD. This may be due in part to the heterogeneity of MDD phenotypes, and complex polygenicity [15]. Therefore, novel and creative approaches may be needed in order to identify genetic contributions to MDD in both adults and pediatric populations—some of which will likely be shared between the two populations.

Therefore, future research should focus on establishing a better understanding of the molecular mechanisms that contribute to MDD, the identification of subgroups of pediatric MDD that may respond better to different therapeutics, and the development of pharmacological agents that can specifically target these pathways. There are currently nine therapeutic trials ongoing in the U.S. to investigate the efficacy and safety of antidepressants in pediatric MDD populations [54]. Use of animal preclinical pediatric MDD models may allow for early identification of ineffective therapeutics in pediatric populations, so that greater focus can be given to the best possible therapies.

The current best practice for the treatment of adolescent MDD couples psychotherapy with pharmacotherapy. This combination therapy increases efficacy and produces earlier remission timelines, while reducing the risk of suicide [17]. If psychotherapy is not available and SSRI treatment is used alone, weekly checkups for the first month and every other week for the second month of treatment are recommended [34]. Options for the treatment of MDD in children remain limited, and we eagerly await advancements made in the pathophysiology and treatment of depression for this age group. Each advancement in the prevention, diagnosis, and treatment of pediatric MDD will continue to move us closer to providing the best care possible to depressed pediatric patients, in the hope of alleviating their symptoms.

## 6. Literature Selection

An outline of the specific topics relevant to the treatment of pediatric MDD was created by the authors and relevant literature was searched using PubMed and Google Scholar as well as by researching websites for the following reference organizations: Centers for Disease Control, World Health Organization, Diagnostic and Statistical Manual 5 (DSM5), U.S. Preventative Task Force, ClinicalTrials.gov and U.S. Food and Drug Administration (FDA).

## Figures and Tables

**Table 1:** Symptoms for the Diagnosis of Major Depressive Disorder (MDD). There are nine main categories of symptoms that are used for the diagnosis of MDD (column 1) and some qualifications that are specific to pediatric patients (column 2).

Symptoms of MDD	Symptoms Specific for Pediatric Patients
Depressed mood	May present as irritable mood
Decreased interest or pleasure	
Significant weight change	May include failure to meet expected weight gain
Altered sleeping habits	
Altered psychomotor activity	
Fatigue/loss of energy	
Feelings of worthlessness/feelings of guilt	
Inability to concentrate and think	May include significant drop in grades or not maximizing educational potential
Suicidal ideation	

**Table 2:** Treatments for Pediatric MDD. The two main categories of therapeutic approaches for pediatric MDD treatment are psychotherapy (CBT and IPT) and psychopharmacology (SSRIs: Fluoxetine and Escitalopram). These treatments are indicated for different age ranges, with potential concerns for the SSRIs. MDD: Major Depressive Disorder; CBT: cognitive behavioral therapy; IPT: Interpersonal Therapy; SSRI: selective serotonin reuptake inhibitors.

Treatment	Indicated for	Limited Evidence for	Potential Concerns
Psychotherapy			- Availability of trained clinicians
CBT	- Above 13 years	- Below 13 years	
IPT	- Above 13 years	- Below 13 years	
Pharmacological SSRIs			- Possible increased suicide risk - Possible induction of mania in unrecognized bipolar disorder
Fluoxetine	- 8–18 years	- Below 8 years	
Escitalopram	- 12–17 years	- Below 12 years	
